# When the Numbers Lie: Uncovering Pseudohyponatremia After an Anabolic Steroid Injection

**DOI:** 10.7759/cureus.98516

**Published:** 2025-12-05

**Authors:** Danielle C Lacasse, Ashley M Otto, Nathan Bosse, Matthew F Baker

**Affiliations:** 1 Internal Medicine, Naval Medical Center Portsmouth, Portsmouth, USA; 2 Nephrology, Naval Medical Center Portsmouth, Portsmouth, USA

**Keywords:** anabolic steroid, general nephrology, laboratory finding, pseudo-hyponatremia, serum osmolality

## Abstract

Pseudohyponatremia is a laboratory artifact in which serum sodium levels appear falsely low despite normal serum osmolality. It typically occurs in the presence of hypertriglyceridemia or paraproteinemia, but this case highlights a rare etiology: inadvertent intravenous injection of an oil-diluted anabolic steroid. A 42-year-old healthy man presented to the emergency department with nausea, vomiting, and presyncope. He reported injecting trenbolone intramuscularly three days prior and noted blood aspiration before injection, suggesting possible intravenous administration. He denied other recent illnesses or exposures. On examination, he was afebrile, hemodynamically stable, and non-toxic, with an unremarkable physical exam. Initial laboratory results revealed a serum sodium level of 125 mmol/L. However, the measured serum osmolality was 289 mOsm/kg while the calculated osmolality was 259 mOsm/kg, revealing an osmolal gap. A point-of-care iSTAT revealed a sodium level of 137 mmol/L, indicating true normonatremia and supporting the diagnosis of pseudohyponatremia. Additional labs showed erythrocytosis, consistent with anabolic steroid use, and no evidence of hypertriglyceridemia or paraproteinemia. Nephrology was consulted due to concern for symptomatic hyponatremia. With a normal measured osmolality, normal lipid and protein levels, and a history of recent anabolic steroid injection, the low serum sodium level was attributed to a laboratory artifact resulting from nonaqueous solvent interference. The patient was admitted for observation and remained stable, with improving symptoms and normalizing labs. Serum sodium by main-lab indirect analysis was 136 mmol/L by the time of discharge on hospital day two. He was discharged with outpatient follow-up. In patients with unexplained hyponatremia and normal clinical status, pseudohyponatremia must be considered. Prompt recognition can prevent unnecessary treatments, reduce patient risk, and guide appropriate care.

## Introduction

Hyponatremia is a common laboratory abnormality in hospitalized patients [1]. Evaluating serum tonicity is critical, as the management of hyponatremia depends on whether the condition is hypotonic, isotonic, or hypertonic. Symptomatic hypotonic hyponatremia can be a medical emergency necessitating prompt recognition of the condition and the use of hypertonic saline to prevent brain herniation [2].

Before initiating treatment, it is essential to exclude causes of normotonic and hypertonic hyponatremia. Pseudohyponatremia is a laboratory artifact defined by a serum sodium concentration <135 mEq/L in the setting of normal serum osmolality (275-295 mOsm/kg), typically resulting from displacement of plasma water by elevated concentrations of lipids or proteins [3,4]. Hypertonic hyponatremia, by contrast, occurs when serum sodium is low in the presence of elevated serum osmolality (>295 mOsm/kg), most often due to the presence of osmotically active solutes such as glucose or mannitol [4].

When pseudohyponatremia is suspected, identifying the underlying etiology is essential. It is classically associated with severe hyperlipidemia or hyperproteinemia and has also been reported in patients receiving intravenous immunoglobulins, total parenteral nutrition, or lipid-based infusions [3,5].

Anabolic-androgenic steroids (AASs) are synthetic derivatives of testosterone that are frequently misused for performance enhancement and muscle mass augmentation. According to the 1994 National Household Survey on Drug Abuse, approximately 1.1 million Americans reported having used anabolic steroids, highlighting the widespread prevalence of non-medical use [6]. While the cardiovascular, hepatic, endocrine, and psychiatric complications of AAS abuse are well-documented, emerging case reports describe a broader range of systemic effects, including pulmonary, renal, and metabolic derangements. For example, a 42-year-old bodybuilder developed acute respiratory distress syndrome, acute kidney injury, and refractory supraventricular tachycardia after chronic AAS use, requiring extracorporeal membrane oxygenation, continuous veno-venous hemodialysis, and catheter ablation. Prolonged steroid use was believed to contribute to multi-organ dysfunction through immunomodulatory and pro-inflammatory mechanisms [7].

We present a case of pseudohyponatremia secondary to inadvertent intravenous injection of an oil-diluted anabolic steroid in an otherwise healthy patient. To our knowledge, pseudohyponatremia resulting from intravenous injection of an oil-based anabolic steroid has not been previously reported in the literature. This case underscores the importance of including AAS use in the clinical history, especially in patients presenting with unexplained multiorgan involvement. Recognition of AAS-related complications is critical, as it may influence diagnostic reasoning, disease progression, and clinical outcomes.

## Case presentation

An otherwise healthy 42-year-old civilian male presented to the emergency room with nausea, vomiting, and presyncope. He denied any chest pain, shortness of breath, abdominal pain, or diarrhea. He had recently administered an unknown amount of trenbolone (an anabolic steroid) intramuscularly three days prior. When aspirating the syringe before injecting the steroid into his buttock, he saw a return of bright red blood into the syringe and injected the steroid despite this. His biggest concern was that this was the cause of his presenting symptoms; otherwise, he had no recent illness, work exposures, or lifestyle changes.

Upon presentation in the emergency department, the patient was afebrile with stable vital signs (temperature 36.4°C, blood pressure 149/81 mmHg, pulse 71 bpm, respiratory rate 16/min, and SpO₂ 97% on room air). The physical examination was unremarkable; the patient appeared non-toxic and showed no signs of volume depletion or overload, neurologic deficit, or respiratory distress. 

Initial laboratory values showed a serum sodium of 125 mmol/L, raising concern for symptomatic hyponatremia. However, the measured serum osmolality was 289 mOsm/kg with a calculated osmolality of 259 mOsm/kg. This discordance raised suspicion of pseudohyponatremia. A point-of-care chemistry panel was then ordered, revealing a sodium level of 137 mmol/L, which confirmed the diagnosis of pseudohyponatremia. Lipid panel (cholesterol - 165 mg/dL; reference range: <200, HDL cholesterol 21 mg/dL; reference range: >60, LDL- 111 mg/dL; reference range: <130, triglycerides 163 mg/dL; reference range: <150) and serum protein studies (Gamma Globulin LC- 0.8 g/dL; reference range: 0.4-1.8, M-Spike - not observed, Globulin total LC- 2.7 g/dL; reference range: 2.2-3.9, immunofixation result LC- unremarkable) were unremarkable. 

With no evidence of hyperlipidemia or paraproteinemia, pseudohyponatremia was attributed to an exogenous nonaqueous substance introduced by the recent injection. The patient was admitted for observation due to the unknown nature of the steroid preparation and potential for delayed complications. Over the next 24 hours, his symptoms improved, and he remained hemodynamically stable without evidence of neurologic or respiratory compromise. On hospital day two, his serum sodium had normalized to 136 mmol/L. He was discharged with instructions for outpatient follow-up.

## Discussion

This case highlights a novel cause of pseudohyponatremia involving intravenous administration of an anabolic steroid. Routine laboratory sodium measurements rely on indirect ion-selective electrode (ISE) methods, which involve diluting plasma and are based on the assumption that 93% of the plasma volume is water [1,2]. This technique is generally accurate, but in the presence of abnormal concentrations of serum proteins, lipids, or exogenous substances, the proportion of plasma water is reduced [2,3]. As a result of this calculation based on a false assumption, serum sodium may be reported as falsely low despite true normonatremia and normal serum tonicity. In such scenarios, distinguishing true hypotonic hyponatremia from a laboratory artifact becomes essential to avoid patient risk from unnecessary medical interventions. 

Comparing measured and calculated serum osmolality plays a key role in this regard. The serum osmolality reflects the concentration of all solutes in plasma, including sodium, glucose, urea, alcohols, etc. This is measured through freezing point depression or vapor pressure methods. Calculated osmolality, on the other hand, estimates osmotic pressure based on sodium, glucose, and blood urea nitrogen, assuming these are the principal solutes. In pseudohyponatremia, the measured osmolality remains normal, as water-based solutes are unchanged; however, the calculated osmolality appears low due to falsely depressed sodium values. This osmolar gap suggests the presence of unmeasured substances interfering with standard sodium measurement [2]. In our case, prompt recognition of this gap and the diagnosis of pseudohyponatremia avoided further medical interventions such as hypertonic saline commonly used to treat symptomatic hyponatremia. 

In this case, the patient had no evidence of hyperlipidemia or paraproteinemia, ruling out the most common causes [3]. Instead, he had a recent history of accidentally injecting an oil-based anabolic steroid compound intravascularly, introducing nonaqueous material into the bloodstream [5,6]. Many unregulated steroid preparations are suspended in oil-based solvents, such as cottonseed oil, sesame oil, or castor oil, or contain alcohol-based diluents, like methanol or ethanol, to increase solubility and bioavailability [8,9]. These nonaqueous agents alter plasma composition, displacing water and interfering with indirect ISE readings, ultimately resulting in pseudohyponatremia. In contrast, a direct ISE method, such as those used in blood gas analyzers, assesses sodium activity without dilution and is unaffected by abnormal lipid or protein levels. For our patient, the point-of-care sodium reading of 137 mmol/L confirmed true normonatremia, despite a routine lab sodium of 125 mmol/L, indicating a laboratory artifact. Recognizing this discrepancy, alongside normal osmolality and clinical stability, prevented unnecessary interventions and allowed for safe observation.

## Conclusions

This case illustrates an unexpected cause of pseudohyponatremia and anabolic steroid use and reemphasizes the diagnostic value of integrating clinical findings with osmolality data and direct sodium measurement. In patients with unexplained hyponatremia and normal clinical status, pseudohyponatremia must be considered. Prompt recognition and confirmation with direct ISE methods can prevent unnecessary treatments, reduce patient risk, and guide appropriate care.
